# Role of sex and sex hormones in PD-L1 expression in NSCLC: clinical and therapeutic implications

**DOI:** 10.3389/fonc.2023.1210297

**Published:** 2023-10-24

**Authors:** Vianey Rodriguez-Lara, Giovanny Soca-Chafre, Maria Rosa Avila-Costa, Juan Jose Juarez-Vignon Whaley, Jeronimo Rafael Rodriguez-Cid, José Luis Ordoñez-Librado, Emma Rodriguez-Maldonado, Nallely A. Heredia-Jara

**Affiliations:** ^1^ Department of Cell and Tissue Biology, Faculty of Medicine, UNAM, Mexico City, Mexico; ^2^ Oncological Diseases Research Unit (UIEO), Hospital Infantil de México Federico Gómez, Mexico City, Mexico; ^3^ Neuromorphology Laboratory, Facultad de Estudios Superiores Iztacala, UNAM, Mexico City, Mexico; ^4^ Department of Thoracic Oncology, Instituto Nacional de Enfermedades Respiratorias, Mexico City, Mexico; ^5^ Traslational Medicine Laboratory, Research Unit UNAM-INC, Instituto Nacional de Cardiología Ignacio Chávez, Mexico City, Mexico

**Keywords:** NSCLC, PD-1/PD-L1 pathway, immunotherapy, estrogen, androgen

## Abstract

Currently, immunotherapy based on PD-1/PD-L1 pathway blockade has improved survival of non-small cell lung cancer (NSCLC) patients. However, differential responses have been observed by sex, where men appear to respond better than women. Additionally, adverse effects of immunotherapy are mainly observed in women. Studies in some types of hormone-dependent cancer have revealed a role of sex hormones in anti-tumor response, tumor microenvironment and immune evasion. Estrogens mainly promote immune tolerance regulating T-cell function and modifying tumor microenvironment, while androgens attenuate anti-tumor immune responses. The precise mechanism by which sex and sex hormones may modulate immune response to tumor, modify PD-L1 expression in cancer cells and promote immune escape in NSCLC is still unclear, but current data show how sexual differences affect immune therapy response and prognosis. This review provides update information regarding anti-PD-1/PD-L immunotherapeutic efficacy in NSCLC by sex, analyzing potential roles for sex hormones on PD-L1 expression, and discussing a plausible of sex and sex hormones as predictive response factors to immunotherapy.

## 1 Introduction

Lung cancer (LC) holds the highest cancer-related incidence and mortality worldwide and is expected to reach 3.2 million deaths globally in 2050 (1). The LC prognosis after diagnosis remains poor, and the 5-year survival rate is less than 20% (2). LC is classified into small (SCLC, 15%) and non-small types (NSCLC, 85%) (3–5). NSCLC exhibits several differences by sex; since women are frequently non-smoker, diagnosed at younger age, and present adenocarcinoma with EGFR mutations. Women also respond better to chemotherapy and men to immunotherapy, whereas outcomes and survival are significantly better in women (4, 6–8). Furthermore, NSCLC is influenced by sex hormones, mainly estrogens (4, 9).

Targeted and immune therapies have increased LC patients’ survival (10). Median overall survival (OS) for chemotherapy is less than a year, while combined with immunotherapy, OS almost doubles (11). PD-1/PD-L1 based immunotherapy improves NSCLC survival, however sex-derived differences have been reported, suggesting sex as a potential predictor for immunotherapy response (12–14). Sex hormones regulate immune response modifying PD-L1 expression, however in LC this relation is still being explored. This article focuses on PD-1/PD-L1 NSCLC immunotherapy, discussing sex differences in response to PD-L1 blockade, as well as sex-related effects and sex hormones impact on the PD-1/PD-L1 pathway and therapeutic responses implications.

## 2 PD-1/PD-L1 pathway

PD-1 is a transmembrane protein from the CD28/CTLA-4 immunoglobulin family expressed on different immune cells. PD-1 controls immune responses and T-cell activation, proliferation, and effector activity by binding PD-L1/2. Cancer cells inactivate T-cells and accomplish immune evasion through PD-L1 expression (15, 16). Intrinsic PD-L1 regulation includes genetic (transcriptional regulation through KRAS, EGFR, ALK pathways) and epigenetic factors (DNMT1, HDAC, miR-135). Conversely, cytokines (INF-γ), growth factors (EGF, VEGF), hypoxia, post-translational modifications (phosphorylation, glycosylation, palmitoylation, ubiquitination), and even treatments including chemotherapy, radiotherapy, and tyrosine kinase inhibitors, extrinsically modify PD-L1 expression (16, 17).

Among several immune evasion mechanisms, tumor PD-L1 expression alone induces immune escape, inactivating cytotoxic T-cells (18). Therefore, this pathway is an important therapeutic target for multiple cancers including NSCLC, since PD-1/PD-L1 blockade restores immune response increasing patient survival. To date, six PD-1/PD-L1 inhibitors have been approved including nivolumab, pembrolizumab, cemiplimab (anti-PD-1), atezolizumab, durvalumab, and avelumab (anti-PD-L1) (19).

## 3 LC treatment options and PD-1/PD-L1 blockade immunotherapy

LC diagnosis and treatment has developed substantially over the last decade, improving OS, progression-free survival (PFS), treatment response, and quality of life. NSCLC patients undergo EGFR, KRAS and ALK genes mutation. Unfortunately, not all patients are targeted therapies candidates, and may appear mutations resistance and recurrence. In this context, immune PD-1/PD-L1 inhibitors, have completely changed NSCLC management.

Baseline PD-L1 levels stratifies patients with a potentially better response. A higher PD-L1 tumor proportion score (TPS) correlates with improved outcomes. Among NSCLC patients with PD-L1 ≥ 50% treated with pembrolizumab, those with 90-100% PD-L1 TPS show better response (20).

For patients with elevated PD-L1 (≥50%), treatment includes immunotherapy as monotherapy, chemoimmunotherapy, or dual immunotherapy. Those with PD-L1 ≥ 50% without EGFR/ALK mutations who received pembrolizumab had greater OS compared with chemotherapy (30 *vs* 14.2 months) (21). Pembrolizumab also resulted in longer OS compared to other PD1-/PD-L1 inhibitors (26.3 vs. ≤14 months). Additionally, pembrolizumab improved OS combined with chemotherapy and radiotherapy (22, 23). Dual immunotherapy has exhibited durable benefits in OS and PFS regardless of PD-L1 expression compared to chemotherapy (24, 25). Combined immunotherapy or dual immunotherapy might also increase adverse effects (AE) (26).

Moreover, PD-L1 blockade has improved OS and PFS regardless of PD-L1 levels. Low PD-L1 (1-49%) cases are treated with immunotherapy + chemotherapy or dual immunotherapy (25, 27). More patients reached 12-months OS in pembrolizumab plus chemotherapy compared to the placebo (69.2% *vs.* 49.9%) irrespective of PD-L1 levels (28). Similarly, the IMpower 150 showed atezolizumab + chemotherapy increased OS and PFS independently of PD-L1 levels (29).

Finally, patients with negative (<1%) PD-L1 are still candidates for combined immunotherapy with chemotherapy or targeted therapy and dual immunotherapy (25, 30, 31). PD-L1 blockade has significantly improved clinical outcomes mainly in patients with higher PD-L1 levels. However, PD-L1inhibitors are considered the choice treatment even in those without PD-L1 expression, making these agents the LC new gold standard therapy.

## 4 Sex-related differences in response to PD-1/PD-L1 blockade in NSCLC

Although PD-1/PD-L1 blockade improved survival compared to chemotherapy and targeted therapy, sex-related differences have been reported (13, 14, 32). A systematic review (11,351 patients; 67% men and 37% women) showed different ICI efficacy by sex in melanoma and NSCLC. The pooled OS hazard ratio (HR) of ICI treatment was higher for women (12). Moreover, 4 NSCLC trials (1,672 patients; 73.2% men and 26.8 women) evaluated pooled OS-HR of PD1/PD-L1 ICI *vs* chemotherapy, resulting higher risk for females (13) Women also experience more immunotherapy AE (33).This data suggests a significant benefit of ICIs in males. Conversely, women with advanced NSCLC responded better to chemotherapy+PD-1/PD-L1-immunotherapy than men who benefited from PD-L1 blockade monotherapy (14).

A systematic review of trials and observational studies reported improved survival for male patients after pembrolizumab/nivolumab as monotherapy. Otherwise, women experienced increased survival rates, in chemoimmunotherapy (34). Additionally, the pooled HRs comparing ICIs *vs* chemotherapy were 0.74 (95% CI0.67-0.81) for men and 0.83 (95% CI 0.73-0.95) for women (35). Better PFS was also observed in advanced NSCLC male patients treated with ICI (5 months *vs* 4). Nivolumab exhibited significantly higher PFS in males *vs.* females also disease control rate was higher in male (55.7 *vs* 45.7%) and their disease progression was lower (44.3 *vs* 54.3%) (36). All above, supports the increased benefit of ICIs monotherapy for males and ICIs+chemotherapy for female patients.

Contradictory results have also emerged, showing no sex differences in response to immunotherapy as monotherapy or combined. A study involving advanced NSCLC patients treated with ICI monotherapy and ICI+chemotherapy observed no differences in PFS by sex, although differences in prognostic factors were noticed (37). Additionally, no sex-related differences were observed in squamous cell NSCLC patients treated with chemotherapy+PD-L1-inhibitors, although different AE were observed by sex (38).

A higher response to chemotherapy has been reported in women than in men (39, 40). Differences in DNA repair capacity between sexes (41) could explain women’s higher sensitivity to chemotherapy (42). Additionally, chemotherapy might improve immunotherapy by enhancing anti-tumor immune response, recruiting, and activating cytotoxic T-cells, inducing immunogenic cell death, releasing tumor antigens and damage-associated molecular patterns, activating dendritic cells, and reducing T regulatory cells (Treg). But chemotherapy enhancing effects to immunotherapy are produced when administered locally since systemic chemotherapy produces high non-specific toxicity (43, 44). These facts could explain the higher chemotherapy response plus immunotherapy observed in women. Higher sensitivity to immunotherapy as monotherapy in men could be explained by disparities in PD-L1 expression.

Some confounding variables including previous treatments, tumor mutational burden (TMB), and smoking habit could explain the controversial response to ICI by sex. Since, there is a sex bias in NSCLC features, it is critical to elucidate sex effects on immunotherapy responses to improve future therapies.

## 5 Sex-driven distinct PD-L1 expression in NSCLC

Sex determines diverse conditions, including lifestyle and toxicant exposure, as well as genetic, and immune features that modify cancer biomarker expression, promoting significant differences in treatment response, including PD-L1 inhibitors. Ye et al., found differences by sex in immune characteristics impacting NSCLC immunotherapy (45).

Several studies show sex differences in PD-L1 levels, which might explain LC immunotherapy response disparities (46–49). A high percentage of PD-L1 positive NSCLC tumors correspond to men, who exhibit higher PD-L1 TPS than females (48–51). Fu et al., reported 18.3% of women with NSCLC *vs* 26% of men with PD-L1 TPS of 1-49%, and only 5.5% of women *vs* 17% of men with PD-L1 TPS ≥ 50% (49). Lin et al., reported 13.6% of men with high PD-L1 TPS (≥50%) *vs.* only 3.8% in females NSCLC patients (52). These findings have been supported by several studies summarized in **Table 1** (47–55). Conversely, no association between PD-L1 expression and sex has also been reported (56). Despite the discrepancies, accumulating evidence discloses differences by sex in PD-L1 status in NSCLC (40, 42, 44–49).

**Table 1:** Differences in PD-L1 expression by sex in NSCLC patients.

Sex	PD-L1 TPS			Reference
	<1 (%)	1-49 (%)	>50 (%)	
Male	57	26	17	(49)
Female	76.2	18.3	5.5	
Male		26	25	(51)
Female		34	15	
Male	44.5	29.9	25.6	(53)
Female	54.9	30.9	14.2	
Male	59.4	71.4	79.7	(48)
Female	40.5	28.5	20.3	
Male		55.9		(50)
Female		44.1		
Male			13.6	(52)
Female			3.8	
Male	>30		>10	(54)
Female	< 20		< 5	
Male	64.81			(46)
Female	35.19			
Male	36	35	28	(47)
Female	37	31	32	
Male	49.4	16.7	7.73	(55)
Female	17.9	5.5	20.6	

Some intrinsic and extrinsic sex factors might drive differences in PD-L1 levels. Smoking status, generally associated with LC male patients, has been related to PD-L1 expression. High PD-L1 TPS was correlated with smoking history and better immunotherapy response. Smoking patients presented higher and prolonged OS and PFS in ICI *vs.* chemotherapy (57–62). KRAS mutation and squamous histology associate with PD-L1 expression, and tobacco smoking could partially explain differences in PD-L1 levels in NSCLC patients by sex (63). Further studies are needed to confirm sex differences in PD-L1 levels and factors affecting its expression. More women must be integrated into studies, being generally underrepresented. Also, TMB, histology, smoking status, and hormonal factors should be considered.

Steroids sex hormones participate in several carcinogenic pathways in LC and could probably play a role in sex PD-L1 disparities (64, 65). Although many LC patients exhibit low sex hormone levels (mainly estrogen) due to age and menopause, lung tumors produce sex hormones locally through aromatase (ARO) overexpression (66–69). ARO and hormone receptors could modify PD-L1 expression regardless of sex and hormonal status.

## 6 Role of steroid sex hormones in PD-L1 expression

### 6.1 Estrogens in NSCLC

The estrogen pathway has taken relevance in NSCLC given its role in lung carcinogenesis. Estrogen receptor (ER)-β, the most common LC isoform and ARO expression, correlate with poor prognosis and survival (68). ERβ is overexpressed in 60-80% of male and female NSCLC patients. Estrogen (E2), through its nuclear receptors (ERα/ERβ) and G-protein-coupled estrogen receptor (GPER), promotes LC progression by cell proliferation, apoptosis resistance, angiogenesis, epithelial mesenchymal transition (EMT), cell migration and metastases (4, 9, 70, 71). Moreover, an important role for estrogen related receptor alpha (ERRα) has been reported in NSCLC, which stimulates proliferation and EMT (72, 73).

E2 also modifies tumor microenvironment through pro-inflammatory cytokines and recruiting Tregs promoting immune evasion (74). Additionally, E2 up-regulates chemokine receptor CXCR4, contributing to immune evasion and metastases in NSCLC (75, 76). Currently the role of E2 in immune evasion and PD-L1 control in LC is being explored.

#### 6.1.1 PD-L1 regulation by estrogen pathway in cancer

Estrogens downregulate PD-L1 expression in endometrial and breast cancer (BC) and correlates with ER-negative status in BC (77, 78). In MCF-7 cells, E2 negatively regulated PD-L1 transcription (79). Moreover, antiestrogens increased PD-L1 expression in ER+ BC (80). E2 probably decreases PD-L1 expression through IL-17 signaling (77). Also, E2/GPER pathway downregulated PD-L1 through COP9-signalosome subunit 5 degradation, as reported in melanoma and pancreatic ductal adenocarcinoma (81, 82).

Paradoxically, PD-L1 expression correlated with ER+, PR+, and Ki67+ in BC (83). E2/ERα increased PD-L1 but not PD-L2 expression in endometrial and BC. PD-L1 expression may be controlled through the PI3K/AKT pathway and post-transcriptional PD-L1-mRNA stabilization in BC (84). In metastatic renal cell carcinoma nivolumab increased E2 levels in male patients (85). Decreased PD-L1 levels by nivolumab increase IL-6 in melanoma animal models, consequently, increasing E2 synthesis and promoting immune evasion (85–87).

In melanoma and prostate cancer (PC), estrogen receptor modulators (SERMs) have been suggested to improve immunotherapy (88, 89). Besides, SERMs and degraders (SERDs) significantly improved immunotherapy efficacy in BC, suggesting an E2 role in up-regulated PD-L1 (90).

**Figure 1:**
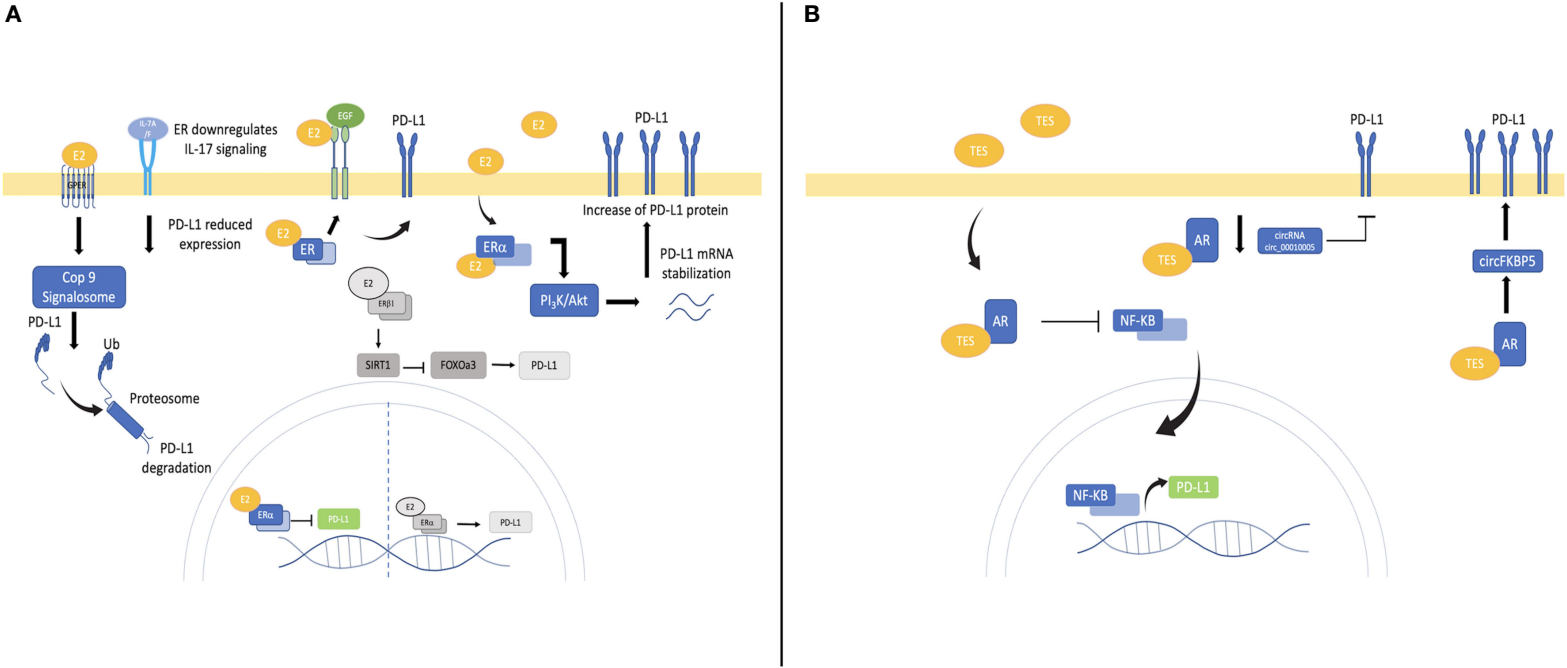
Mechanisms involved in PD-L1 control by estrogen and androgen in cancer and NSCLC. **(A)** Estrogen pathway downregulates PD-L1 by repressing its transcription, promoting its proteosomal degradation and IL-17 downregulation. E2/ER also activates the PI3k/Akt pathway promoting RNA stabilization and increasing PD-L1 protein as observed in breast cancer. E2/ER through EGFR/EGF pathway might stimulate PD-L1 increase in lung cancer. Emerging mechanisms by E2 pathway might up-regulates PD-L1 in NSCLC are represented in gray. E2/ERα increases PD-L1 transcription. Moreover, E2/ERβ activates SIRT1 promoting FOXOa3 degradation and consequently PD-L1 increases **(B)** Androgen pathway downregulates PD-L1 transcriptionally, mainly by inhibiting NF-kB translocation and decreasing promoter activation. AR regulates PD-L1 expression post-transcriptionally by modifying circRNAs.

Estrogen mechanisms modifying PD-L1 seem to be complex and may depend on several factors such as cancer type, histology, TMB, ER isoforms, ARO expression, estrogen levels, and microenvironmental features (**Figure 1**). This relationship needs to be explored since E2 pathway blocking could improve immunotherapy in some cancers, including NSCLC.

#### 6.1.2 PD-L1 and estrogen pathway in NSCLC

The E2 role in NSCLC immune evasion has been scarcely investigated, and its PD-L1 relationship is emerging. For instance, E2 reduced cytotoxic lymphocyte activity by inducing ERβ1/5. Also, E2 up-regulated PD-L1 by increasing ERβ/SIRT1, Snail transcriptional factor while reducing FOXO3a (91). ERβ could be a critical target to improve immunotherapy given its higher expression in male and female NSCLC patients.

E2/ERα increased PD-L1 transcription was recently reported *in vitro*. Additionally, *in vivo*, letrozole (ARO inhibitor) improved pembrolizumab efficacy, while in NSCLC patients, ERα was a predictive response factor to pembrolizumab, even stronger than sex and PD-L1 levels (92). This could be explained by high ER expression independently of sex in NSCLC (9). Thus, ER could become a biomarker to predict to immunotherapy response in NSCLC.

Decreased levels of the receptor for advanced glycation end products (RAGE) associate with lung carcinogenesis and metastasis regulating PI3K/AKT and KRAS-RAF1 signaling. RAGE participates in redox regulation, and its polymorphisms are linked to LC incidence and progression (93). Thus, RAGE is an important axis in LC development. Recently it was reported that HMGB-RAGE promotes PD-L1 expression in BC (94). Also, E2 treatment up-regulated RAGE in human microvascular endothelial cells (94). The association between E2, RAGE and PD-L1 in NSCLC has not been elucidated; however, it could represent a key mechanism underlying carcinogenesis and immune evasion.

Besides, estrogens could modify PD-L1 in NSCLC through the EGFR pathway. EGFR/EGF activation increases E2 through ARO up-regulation (9, 67, 95). Since EGF enhancing PD-L1 in NSCLC (96), E2 could stimulates PD-L1 through the EGF/EGFR pathway; however, this hypothesis needs to be tested.

Differences in serum PD-1 (sPD-1) by sex were reported in NSCLC patients, where females exhibited higher sPD-1 and PD-1 on CD4+ T cells. Increased testosterone levels were also reported (97) suggesting sex hormones could control PD-1.

All these data support E2 contribution to immune evasion up-regulating PD-L1 through diverse mechanisms involving both ERα/ERβ in NSCLC (**Figure 1**). Antiestrogens could improve immunotherapy even in low PD-L1 conditions due to high ER expression in NSCLC. This is a new approach showing how estrogen pathway promotes lung carcinogenesis and how antiestrogens could improve immunotherapy as well as targeted therapy. However further studies are warranted to explore these mechanisms and their potential therapeutic impact.

### 6.2 Androgens in NSCLC

LC androgen participation is still poorly explored and contradictory. Androgen receptor (AR) is downregulated in NSCLC tissues and cell lines, without differences by sex and staining. Higher AR levels associate to better survival rates. miR-224-5p is up-regulated in NSCLC promoting proliferation, decreased apoptosis, migration, and metastasis by, downregulating AR (98). Furthermore, AR+ status relate to favorable OS in NSCLC metastatic disease (99), not in early stages (100).

On the other hand, AR was overexpressed mainly in NSCLC male patients (101). AR was detected in 20% of LC patients; higher levels were in advanced LC stages associated with progression and metastasis (102). Moreover, targeting androgen pathway in NSCLC patients resulted in better survival (103), and reduced risk to second primary LC for PC patients (104). Androgen deprivation therapy (ADT) for PC, improved survival in NSCLC after diagnosis, particularly in Caucasians (105). *In vitro*, androgen up-regulated gene expression involved in DNA repair, oxygen transport, apoptosis, and hemoglobin synthesis while downregulated CYP1A1 (106). Also, AR promotes proliferation through cyclin D1 regulation, stimulate migration and invasion and regulates OCT-4 protein supporting stemness (101, 107, 108). Finally, KRAS mutational profiles are linked to AR levels in NSCLC (109). Despite controversial data, androgen pathway apparently plays an important role in lung carcinogenesis highlighting its therapeutic potential.

#### 6.2.1 Androgen pathway and PD-L1 regulation

Although men appear to respond better to immunotherapy in NSCLC, androgen activity on immune response, evasion mechanisms and PD-L1 expression in LC has not been elucidated. However, AR down-regulates PD-L1 across different malignances.

Inverse correlation between AR and PD-L1 levels has been reported in muscle invasive or metastatic urothelial (110), thyroid (111) and hepatocellular carcinomas (112), suggesting PD-L1 downregulation through the AR pathway. In thyroid cancer, dihydrotestosterone reduced PD-L1 in a time- and dose-dependent manner, while flutamide (AR antagonist) restored PD-L1 expression. AR could decrease PD-L1 expression inhibiting NF-kB nuclear translocation and reducing PD-L1 promoter activation (111). In hepatocellular carcinoma AR downregulates PD-L1 acting as PD-L1 transcriptional repressor (112). In contrast, in bladder cancer targeting AR enhances NK activity decreasing PD-L1 expression; both anti-androgen treatment and knockdown significantly reduced PD-L1 expression and stimulated NK cell-mediated bladder cancer cell death by downregulating circRNA circ_0001005 (113). Also, Tang and coworkers (114) demonstrated how dihydrotestosterone/AR higher dose increased PD-L1 expression and suppressed NK cells immunotherapy efficacy in castration- resistant PC cells (CRPC) (**Figure 1**). AR-blockade improved sex-bias BRAF/MEK-targeted therapy response in melanoma (115), and enhanced CD8/T-cells activity in CRPC improving PD-1/PD-L1-inhibitors response (116), suggesting that AR promote targeted and immunotherapy resistance, and shows sex impact in treatment.

Although androgen immunosuppressive effects have been documented, and ADT improves PC immunotherapy (117), its relationship with PD-L1 in clinical and experimental conditions remains contradictory. Future studies are necessary to clarify androgen´s impact on PD-L1control in NSCLC, since PD-L1 is a key target in immunotherapy, to which men appear to respond better.

## 7 Conclusion and perspectives

NSCLC is a significantly different disease between women and men, influenced by sex hormones. The estrogen and androgen roles in NSCLC immune response is not completely understood. Currently, data remain contradictory on differential response to PD-L1-based immunotherapy sex-related. Nevertheless, several studies show higher benefit in male NSCLC patients which could be explained by higher PD-L1 levels. Sex could be a predictive response factor to NSCLC immunotherapy; however, sex-derived differences must be validated as well as consistency across different populations, equilibrated groups by sex, histological subtypes, mutational profiles, and smoking status. Additionally, women should be stratified by hormonal status and serum hormonal levels could be measured to clarify the sex and sex hormones impact on PD-L1 control and immunotherapy responses.

Some factors sex-associated as TMB and tobacco smoking modify PD-L1 which partially explains immunotherapy differential responses. Hormones, mainly estrogen also affect the PD-L1 pathway in NSCLC. Although PD-L1 control by E2 remains controversial in different cancers; in NSCLC emerging data shows E2/ER up-regulates PD-L1 suggesting that SERDs might enhance NSCLC immunotherapy response. Studies on sex and sex hormones effects in immune evasion are critical, since antihormonal therapy might be easily extrapolated to NSCLC treatment, but a wide gap still exists in this field. Androgen effect on immune evasion mechanisms through PD-1/PD-L1 in NSCLC remains to be elucidated.

Finally, all this data shows the sex and sex hormones relevance in LC progression and its impact on PD-L1 based immunotherapy response. However, it is essential to strength research on sex-related differences to understand LC behavior, identify biomarkers, predict immunotherapy response, and establish better therapeutic guidelines according to sex and hormonal status.

## Author contributions

VR-L designed, wrote, review, edited the manuscript, made the final version and coordinated teamwork. All authors contributed to the article by writing or final editing of the manuscript. All authors contributed to the article and approved the submitted version.
