# Erratum: Role of sex and sex hormones in PD-L1 expression in NSCLC: clinical and therapeutic implications

**DOI:** 10.3389/fonc.2023.1356381

**Published:** 2024-01-05

**Authors:** Frontiers Production Office

**Affiliations:** Frontiers Media SA, Lausanne, Switzerland

**Keywords:** NSCLC, PD-1/PD-L1 pathway, immunotherapy, estrogen, androgen

Due to a production error, an author name was incorrectly spelled as “Soca-Chafre Giovanny”. The correct spelling is “Giovanny Soca-Chafre”.

The publisher apologizes for this mistake. The original version of this article has been updated.

